# Supplementary material for: VP2 residue N142 of coxsackievirus A10 is critical for the interaction with KREMEN1 receptor and neutralizing antibodies and the pathogenicity in mice
Source: PLoS Pathog. 2023 Oct 3;19(10):e1011662. doi: 10.1371/journal.ppat.1011662 (PMC10547193; doi:10.1371/journal.ppat.1011662)
Supplement: Attachment — Submitted filename: 2023.8.24 Response to Reviewer.docx [file ppat.1011662.s001.docx]

Dear reviewers,

We are grateful for the insightful comments from the reviewers and have carefully considered them. Below is the detailed point-by-point response to the comments. The related changes made in the revised manuscript are highlighted in yellow.

**Response to Reviewer #1’s comments:**

**Summary:**

Reviewer #1: Li et al., reported here the VP2 N142 of CAV10 is essential for viral recognition by KRM1 receptor and also correlates with neutralizing antibodies and pathogenicity. The cellular experiments, rescue experiments, and in vivo experiments conducted in mice collectively offer comparatively substantial data support. Generally, this paper is interesting, however some major issues have to be addressed before accepted by PLoS Pathogens.

**Response:** Thanks for the positive overall evaluation. Below are our responses to the reviewer’s specific comments.

**Major Issues:**

1) The authors also mention in Figure 3D that this mutation is only present in 1 out of 255 sequences in the NCBI database. This largely weakens the significance of this study. I wonder if the authors have investigated the presence of mutations in N2142D in other enteroviruses that rely on KRM1 invasion?

**Response:** Thanks for the comments. It is reported that KRM1 is a host entry receptor for CVA2 to CVA6, CVA10, and CVA12 [1]. By sequence alignment, we found that CVA2, CVA4, and CVA6 have polar amino acid N at position 2142, while CVA3, CVA5, and CVA12 have other polar amino acids (T, S, or Q) at this position. A total of 76 CVA2 VP2 protein sequences, 135 CVA4 VP2 sequences, and 1019 CVA6 VP2 sequences were downloaded from NCBI database (as of August 2023) using BLAST. The analysis showed that all of the CVA2 and CVA4 VP2 sequences had an asparagine (N) at position 2142. For CVA6 VP2 sequences, 1018 had an asparagine (N) at position 2142, while the last one had an arginine (R) at this position. Thus, the N2142D mutation is not present in all of naturally occurring CVA2, CVA4, and CVA6 virus isolates. This is reasonable because N2142D mutation could substantially impair viral fitness, making this mutation relatively uncommon in nature. We have added discussion of this point to the Discussion section (please see lines 376-384), to read “In addition, we investigated the presence of the N2142D mutation in other KRM1-dependent enteroviruses, including CVA2, CVA3, CVA4, CVA5, CVA6, CVA10, and CVA12 [29]. NCBI blast analysis showed that CVA2, CVA4, and CVA6 had a polar amino acid N at position 2142, while CVA3, CVA5, and CVA12 had other polar amino acids (T, S, or Q) at this position. N2142D mutation is not present in all of these naturally occurring virus isolates. This is reasonable because N2142D mutation could substantially impair viral fitness, making this mutation relatively uncommon in nature and extremely difficult to detect.”.

2) Previous studies suggest that the same virus exhibits different titers when infecting different cells due to differences in protein expression levels on different cell surfaces, what is the infectivity of the mutation in N2142 in other enterovirus-sensitive cell lines?

**Response:** Thanks for the insightful comments. We agree with the reviewer's opinion. Since RD cells are commonly used for CVA10 research, we did not investigate infectivity of CVA10 in other cell lines in this study. Due to time and resources constraints, we were unable to carry out this experiment in a short period of time. Instead, we have added discussion of this point to the Discussion section (please see lines 416-425), to read “A limitation of our study is that due to time and resource constraints, we only used RD cells for CVA10 research and did not investigate the infectivity of wildtype and mutant CVA10 viruses in other enterovirus-sensitive cell lines. Previous studies report that CVA10 can exhibit quite different growth kinetics when infecting different human cell lines, such as RD (rhabdomyosarcoma cells), Hela (cervical epithelial cells), HEK 293A (embryonic kidney cells), KB (oral carcinoma cells), SK-N-SH (neuroblastoma cells), and MRC-5 (lung fibroblasts) [33, 34]. This phenomenon is probably due to differences in cell surface expression level of the KRM1 receptor. It will be important in the future to examine whether the N2142A mutation would affect CVA10 viral replication in these human cell lines.”.

3) Furthermore, are there other mutations in nature that are variations favoring virus invasion, which would likely be a trend for further virus epidemic expansion and would provide an early warning scenario for disease epidemics.

**Response:** Thanks very much for the insightful comments. We searched all published articles about CVA10 in the PubMed database up to August 2023. However, no relevant studies were found. Instead, we have added discussion of this point to the Discussion section of the revised manuscript as a new paragraph (please see lines 385-394), to read “In this study, we found a naturally occurring mutation N2142D that is detrimental to CVA10 viral adsorption and infection. We do not study whether there are natural mutations that favor CVA10 viral invasion, but it's a very important scientific question. This kind of research could help predict the virus epidemic trend and provide an early warning for emergence of disease epidemics. For example, in the study of SARS-CoV-2 infection, the N501Y substitution in the Alpha variant spike protein was reported to be able to increase the affinity of viral spike protein to cellular receptor and was proven to be a major determinant of the increased transmission of the Alpha variant [31]. Thus, N501Y variation is believed to be a major adaptive spike mutation of concern [31].”.

**Minor Comments:**

1) Line 75: Species names should be italicized, e.g. Picornaviridae.

**Response:** We have done as suggested in the revised version.

2) Line176-183 & Fig 3C & line 324: The residue N2142 forms hydrogen bonds with D88 and D90 in KRM1 but not with G89. Moreover, the N2142D mutation results in strong repulsive interactions with D88 and D90 in KRM1.

**Response:** Thanks for the comments. We should point out that there are differences in the protein–protein interactions predicted by different software or web servers. We analyzed the CVA10-KRM1 interaction through PISA server. The results of the interaction interface analysis were summarized in Table. R1 (for the reviewers’ view only). The hydrogen bonds were formed between the ND2 atom of CVA10 residue N2142 with the O atom of KRM1 residue D88 and G89, but not with residue D90 in KRM1.

Table. R1. Interaction interface analysis of the CVA10-KRM1 structure (PDB: 7BZU).

| CVA10 | | Distance (Å) | KRM1 | Interaction |
| --- | --- | --- | --- | --- |
| Residue | Location |  | Residue |  |
| T1209 [OG1] | VP1 GH loop | 2.68 | N140 [ND2] | H-bond |
| Q1212 [O] | VP1 GH loop | 3.00 | K141 [NZ] | H-bond |
| T1219 [OG1] | VP1 GH loop | 2.87 | H194 [NE2] | H-bond |
| T2139 [OG1] | VP2 EF loop | 3.10 | Y105 [OH] | H-bond |
| K2140 [NZ] | VP2 EF loop | 3.41 | D88 [OD2] | H-bond, salt bridge |
| K2140 [NZ] | VP2 EF loop | 3.65 | D90 [OD1] | H-bond, Salt bridge |
| N2142 [ND2] | VP2 EF loop | 3.39 | D88 [O] | H-bond |
| N2142 [ND2] | VP2 EF loop | 3.81 | G89 [O] | H-bond |
| E2143 [OE2] | VP2 EF loop | 3.58 | Y66 [OH] | H-bond |

As suggested, we have now modified the sentence “the negatively charged carboxyl groups of the adjacent residues N2142D and D90 of KRM1 may repel each other strongly (Fig. 3C)” (lines 183-185) to “the N2142D mutation may result in strong electrostatic repulsive interactions with the negatively charged carboxyl groups of D88 and D90 in KRM1 (Fig. 3C).”.

3) Fig 5C: The negative stained EM photos in your paper are a bit blurry. Please consider replacing them with clearer ones.

**Response:** Thanks for the comments. We should admit that the previous negative stain electron microscopy images are not clear enough.

To solve this problem, the samples have now been negatively stained **twice** with uranyl acetate to increase the contrast between viral particles and the background. The new electron microscopy images were shown below (new Fig. 4C).

Fig. 4C. The fraction #10 samples of CVA10-WT and CVA10-N142A were diluted to 50 μg/ml and subjected to negative stain electron microscopy. Red and blue arrows indicate full (mature virion) and empty CVA10 particles, respectively.

**Response to Reviewer #2’s comments:**

**Summary:**

Reviewer #2: The authors found a residue in VP2 N142 of CVA10, which is essential to interact with KREMEN1 receptor and neutralizing antibodies and viral virulence in mice. This article provides a better understanding of the molecular mechanisms of CVA10 infection and immunity. At this stage, I have the following suggestions and comments.

**Response:** We thank the reviewer for the positive overall evaluation. Below are our responses to the reviewer’s specific comments.

**Major Issues:**

1) Line 232-238, the author made a conclusion of similar copy number between CAV10 WT and N142A based on the comparable particle number in one micrograph. It is incorrect, because negative stain electron microscopy is not a quantitative tool.

Response: Thanks for the comments. We have now deleted the original Fig. 4D and the corresponding text “the median number of virus particles per EM image was 39 and 45 for CVA10-WT and CVA10-N142A, respectively. Thus, the copy number of virus particles at the same mass concentration was similar between the CVA10-WT and CVA10-N142A groups.”.

2) Figure 4C, the particles in left panel seem solid, corresponding to mature CAV10, while those in the right panel look like empty particles belonging non-infection virus. This should be confirmed.

Response: Thanks for the comments. The CVA10 samples are sucrose gradient fraction samples, leading to the fact that CVA10-N142A viral particles were “positively” stained with uranyl acetate. It is impossible to judge, based on the previous electron microscopy image (also shown below for convenient view), whether the CVA10-N142A particles are solid or hollow.

Fig. 4C. Previous negative stain electron microscopy images of CVA10-WT and CVA10-N142A.

To solve this problem, the virus samples have now been negatively stained **twice** with uranyl acetate to remove sucrose and to increase the contrast between viral particles and the background. Note that the CVA10 samples were previously negatively stained **only once** with uranyl acetate. The new electron microscopy images of CVA10-WT and CVA10-N142A were shown below (new Fig. 4C). From the new images, we can clearly see that the vast majority of the CVA10-WT and CVA10-N142A virus particles are full particles, corresponding to mature virion. We have now modified Fig. 4C and the text accordingly (lines 237-238), to read “As shown in Fig. 4C, ~30 nm spherical particles were observed for both virus samples. And importantly, the vast majority of the CVA10-WT and CVA10-N142A virus particles were full particles, corresponding to mature virion.”.

Fig. 4C. Negative stain electron microscopy images of CVA10-WT and CVA10-N142A.

The fraction #10 samples of CVA10-WT and CVA10-N142A were diluted to 50 μg/ml and subjected to negative stain electron microscopy. Red and blue arrows indicate full (mature virion) and empty CVA10 particles, respectively.

3) Figure 5A, it is hard to make a conclusion of survival assay between CVA10 WT and N142A infection without a control group.

Response: We understand the reviewer's concern. Preliminary experiments showed that the medium control group did not cause any symptoms and death in mice. Moreover, hematoxylin and eosin (H & E) staining showed the limb muscle tissues from medium-injected mice are normal in appearance (Fig. 6A).

In addition, to demonstrate our conclusion that the N2142A mutation can decrease the virulence of CVA10 in mice, it is important to compare the differences in the mortality rate between the CVA10-WT and CVA10-N142A groups, but not the CVA10-N142A and medium-only (control) group. We believe our conclusion is solid enough.

4) The authors harvested tissues from limb muscle, spinal cord, and brain for CVA10 viral load determination. Are there any supportive studies?

Response: Thanks for the comments. A previous study on proliferation characteristics of CVA10 in mice revealed that the viral loads in limb muscle and spinal cord were significantly higher than those in other tissue, including brain, heart, lung, liver, kidney, spleen, and intestine [2], indicating that CVA10 has a strong tropism toward limb muscle and spinal cord. Our data show that viral titers in limb muscle and spinal cord were at least 10 times higher than those in brain (Fig. 5B), in line with the previous report. Thus, tissues from limb muscle and spinal cord should be collected for viral load determination. In addition, brain was also collected and tested as a control, as revealed by the finding that there are no significant correlations between brain viral titers and clinical scores (Fig. 5C).

5) Line 195-205, to analysis the conservation of key sites of CVA10 VP1 VP2 and VP3, the authors align numbers of sequences. The datasets of VP2 and VP3 are much less than VP1, which is unreasonable

Response: Thanks for the comments. We downloaded all CVA10 VP1, VP2, and VP3 sequences from NCBI. The number of CVA10 VP1 sequences is larger than the number of CVA10 VP2 and VP3 sequences. This is probably because VP1 (but not VP2 or VP3) sequence comparison is commonly used for typing of human enteroviruses [3, 4]. VP1 sequence correspond with phylogenetic lineage [4]. Thus, people may prefer to determine the sequences of VP1, but not VP2 or VP3.

**Minor Issues:**

1) Line 121-125, the authors claimed to isolate 10 isolates from well #1, while only 9 mutations were described, why is the last one not mentioned?

Response: Thanks for the comments. As suggested, we have added the information in the result section (lines 125-126), to read “Among the 10 isolates from well #1, 7 had double mutations: N→D at residue 142 of VP2 (N2142D) and N→Y at residue 183 of VP3 (N3183Y), and 2 had an additional mutation at position 1287 (A1287T) or 2156 (T2156S) besides the N2142D+N3183Y mutation, and the last one possessed a double mutation in the VP1 protein (T1219I and S1238G).”

2) In figure 1 B, the names of mutations, such as VP2 N142D and VP3 N183Y, are different from the text and other figures naming N2142D, and N3183Y, respectively, which is not reader-friendly. It should be better to unify the names.

Response: As suggested, we have now unified the names and modified Fig. 1B (also shown below for convenient view).

Fig. 1B. Screening and information of soluble KRM1-resistant mutants. Green circle, no CPE; orange, partial CPE; red, complete CPE. 10 and 9 plaques were isolated from well #1 (the upper well) and #2 (the lower well), respectively, and then sequenced to identify mutations. Viral capsid residues are numbered from 1001, 2001, and 3001 in VP1, VP2, and VP3, respectively.

3) Figure 4E, the units between virus titer and fold change are different, therefore, the table should be modified.

Response: As suggested, we have now modified Fig. 4E (now Fig. 4D, and also shown below for convenient view).

Fig. 4D. The fraction #10 samples of CVA10-WT and CVA10-N142A were diluted to 1 μg/ml and assayed for viral titers. Fold decrease (prefixed with a minus sign) in viral titer is also shown.

4) Figure 4F, it seems more logical to show the three sample in a better order, such as CVA10-WT, CVA-N142A, and Cell only.

Response: As suggested, we have now modified Fig. 4F (now Fig. 4E, and also shown below for convenient view).

Fig. 4E. 100 ng/ml of CVA10 viral particles (fraction #10) were attached to prechilled RD cells at 4 °C for 1 h, and after washing, the levels of CVA10 RNA were determined by RT-qPCR. Data are means ± SD of four replicate samples. The Mann–Whitney test was used to compare differences. *, p < 0.05.

5) Figure 6b is missed in the main text.

Response: Thanks for pointing this out. We have added “Figure 6B” in the revised manuscript (line 308), to read “The CVA10-WT group had a significantly higher mean histopathologic score compared to the CVA10-N142A group (p = 0.0046) (Fig. 6B)”.

Reference

1.	Staring J, van den Hengel LG, Raaben M, Blomen VA, Carette JE, Brummelkamp TR. KREMEN1 Is a Host Entry Receptor for a Major Group of Enteroviruses. Cell Host Microbe. 2018;23(5):636-43 e5. Epub 2018/04/24. doi: 10.1016/j.chom.2018.03.019. PubMed PMID: 29681460.

2.	Gao W, Yue L, Yang T, Shen D, Li H, Song X, et al. Proliferation characteristics of coxsackievirus A10 in mice and immune protection ability of experimental inactivated vaccine. Biomed Pharmacother. 2021;143:112212. Epub 2021/10/16. doi: 10.1016/j.biopha.2021.112212. PubMed PMID: 34649345.

3.	Oberste MS, Maher K, Kilpatrick DR, Flemister MR, Brown BA, Pallansch MA. Typing of human enteroviruses by partial sequencing of VP1. J Clin Microbiol. 1999;37(5):1288-93. Epub 1999/04/16. doi: 10.1128/JCM.37.5.1288-1293.1999. PubMed PMID: 10203472; PubMed Central PMCID: PMCPMC84754.

4.	Oberste MS, Maher K, Kilpatrick DR, Pallansch MA. Molecular evolution of the human enteroviruses: correlation of serotype with VP1 sequence and application to picornavirus classification. J Virol. 1999;73(3):1941-8. Epub 1999/02/11. doi: 10.1128/JVI.73.3.1941-1948.1999. PubMed PMID: 9971773; PubMed Central PMCID: PMCPMC104435.
